# Spatiotemporal characteristics and impact mechanism of high-quality development of cultural tourism in the Yangtze River Delta urban agglomeration

**DOI:** 10.1371/journal.pone.0252842

**Published:** 2021-06-22

**Authors:** Zhangyu Shi, Dehong Xu, Lidi Xu

**Affiliations:** 1 Hangzhou College of Commerce, Zhejiang Gongshang University, Hangzhou, China; 2 School of Tourism and Urban-rural Planning, Zhejiang Gongshang University, Hangzhou, China; 3 Rural Cultural Tourism Promotion Center of Hangzhou College of Commerce, Zhejiang Gongshang University, Hangzhou, China; 4 School of Tourism Management, Hangzhou Polytechnic, Hangzhou, China; Northeastern University, CHINA

## Abstract

The Yangtze River Delta urban agglomeration is the leading and demonstration area for the high-quality development of culture tourism (HDCT) in China. It is of great significance to study the spatiotemporal characteristics and impact mechanism of the HDCT for revealing the internal law of HDCT and promoting the collaborative innovation of culture tourism among cities. Based on the scientific construction of the evaluation system of HDCT, this paper made a quantitative analysis of 26 cities’ HDCT by using coupling coordination degree model, Lisa spatiotemporal transition and spatial Durbin model (SDM). The results show that: The overall level of 26 cities’ HDCT shows a fluctuating upward trend, and presents a "Z" pattern in space. More than 80% of the cities are at the medium and high level. Shanghai has obvious advantages in the primacy degree. There is a significant positive spatial autocorrelation among cities with high-quality of culture tourism development. The spatial clustering and proximity of the same kind are increasing, and the radiation effect is gradually obvious. The local spatial association patterns are mainly HH and LL agglomeration, and the characteristics of polarization are gradually prominent. The local spatial correlation structure of HDCT has strong stability, the transfer inertia between types is prominent, and the overall spatial evolution is lack of integration with obvious path dependence and lock-in effect. The spatiotemporal evolution of the HDCT is a complex process under the interaction of multiple factors, and there is a significant spatial spillover effect (0.256). The level of economic development, technological innovation, professional talent allocation are the three main factors. According to the dominant factor, it can be divided into economy stabilizing type, industry optimizing type, innovation driving type and traffic impacting type. These findings have implications for local governments and tourism management departments to achieve high-quality innovative development of cultural tourism.

## Introduction

Cultural tourism development is a valuable policy in terms of regional economic revitalization and, by extension, of sustainable urban development for improving quality of life [1, 2]. China has entered a stage of high-quality development, in the process of China’s economic development, which is an epoch-making event. China’s tourism industry development has also changed from the single resource development orientation in the early stage to the situation of coordinated and integrated development, from the pursuit of quantity growth to the stage of pursuing quality improvement [3, 4].

Cultural tourism has been the mainstream of the world’s tourism development, and as a major component of international tourism consumption re-affirmed by UNWTO, accounting for over 39% of tourism arrivals. Cultural tourism development has changed from the early single resource development to improving the cultural attraction and competitiveness of tourism destinations, which has become an important driving force for local economic growth and regional coordinated development [3, 5, 6]. The establishment of the Ministry of Culture and Tourism of the People’s Republic of China in 2018 opened a new chapter for the integrated development of Chinese culture tourism from the top-level design. With the remarkable improvement of people’s living standards and the accelerated upgrading of consumption structure, the development quality of cultural tourism, as an industry with strong integration and high knowledge content, has become an important embodiment of the level of regional collaborative innovation [5]. Comprehensively promoting the high-quality development of cultural tourism (HDCT) has become a strategic requirement for China’s cultural tourism industry in the new era [7]. However, at the theoretical level, there is a lack of pioneering thinking and planning on HDCT [8, 9], and there is also a lack of quantitative analysis on HDCT at the practical level.

Improving the quality of tourism development has become an inevitable way for the optimal development of modern service industry [9, 10], which is directly related to the efficiency of regional resource utilization and economic growth [11]. Under the background of culture tourism integration, the high-quality development measurement index of culture tourism should consider the comprehensive index of culture and tourism [6, 12] and a somewhat more suitable method. In the post epidemic era, under the guidance of the new development concept with the domestic big circulation as the main body and the domestic and international double circulation promoting each other, high-quality development is the main keynote for the recovery, revitalization and quality improvement of cultural tourism. Reconstructing the quality evaluation index system, revealing the basic spatiotemporal characteristics and impact mechanism of culture tourism development have strong practical significance for advancing the in-depth integration and promoting the high-quality development of culture tourism.

The possible contributions of this paper are as follows: On the one hand, a scientific index system is very important for evaluation. A large number of literatures have developed different HDCT evaluation index systems, but the results are difficult to compare [8, 13]. The evaluation system constructed in this paper integrates as many cultural industry development related indicators as possible, uses the proportion and growth rate as far as possible to objectively reflect the connotation of "quality" of tourism development, and increases the indicator of cultural tourism integration coordinated development level, which can provide certain reference for the quantitative evaluation of high-quality tourism development in other regions. On the other hand, HDCT has positive spatial autocorrelation and obvious spatial agglomeration characteristics. The strong agglomeration effect and spillover effect of central cities will effectively enhance the high-quality tourism development ability of surrounding cities. Compared with previous studies [6, 13], this is a new discovery that highlights the location information of spatial elements. When studying the spatial characteristics of HDCT, we should not ignore the existence of spatial effect.

## Literature review

The study of cultural tourism has always been one of the focuses of scholars [6, 11]. The existing early researches mainly focused on the classification and theoretical discussion of cultural tourism concepts [11, 12], cultural tourists’ motivation, experience, behavior and their market research [14–16], cultural tourism resources development [5], environmental protection and management of cultural tourism [11, 17].

Quality is the key index to understand the development status of things and evaluate the health and sustainability, which can reflect the improvement of resource utilization or the input and output of factor productivity [18, 19]. In recent years, tourism development quality has been paid more and more attention by experts and scholars, and the research field involves a wide range of content. In a broad sense, the existing literature mainly focused on the following three aspects: Firstly, deeply explaining the governance logic and institutional innovation of HDCT, as well as its interactive with institutional environment [7, 20–21]. Countermeasures and suggestions for the optimization of tourism development quality were put forward [1, 20–22]. Secondly, from the connotation of element quality, establishing an evaluation model to assess the development quality of tourism system elements, mainly including tourism economic quality [23], inbound tourism service quality [24, 25], tourism environment quality [26], tourism experience quality [27], tourism public service quality [28], etc. Thirdly, regarding the evaluation of the development quality or level of tourism destinations, the researches mainly focused on the measurement of investment efficiency of cultural tourism industry [29], the coordinated development of culture tourism industry [30], competitiveness evaluation and regional synergy [13, 31] and the integration situation of culture tourism industry [32].

In a narrow sense, the research on tourism development quality is mainly carried out from the aspects of concept connotation, element system composition and evaluation management [33], such as the comprehensive evaluation of China’s tourism development quality from the aspects of product quality, environmental quality, element quality, industrial growth mode and industrial operation quality [18], the calculation of the quality of tourism economic development based on the three dimensions of efficiency, structure and environment [34], and characteristics and spatial differences of China’s provincial tourism development quality from the perspective of coordinated development of "quality" and "quantity" [19].

Generally speaking, the quality of cultural tourism development has become the frontier of tourism research. However, the existing research still stays on the qualitatively theoretical analysis and static quality level measurement of HDCT, and lacks the regularity summary of its implied spatial characteristics and spillover effect. The dynamic evolution of HDCT spatiotemporal path and influencing mechanism have not attracted enough attention.

The integration of the Yangtze River Delta has become a national strategy, and cultural tourism cannot be separated from its own advantages and functions in promoting the implementation of this new national strategy [9]. It is a major task for the cultural tourism industry in the Yangtze River Delta to promote the high-quality development of culture tourism, accelerate deep integration and integration of culture and tourism, and better support and serve the national strategy. The HDCT urgently needs to establish more scientific measurement methods, clarify the existing quality level and differences, and carry out attribution analysis of regional linkage characteristics. Therefore, taking 26 prefecture-level cities in the Yangtze River Delta urban agglomeration as an example, this paper explores the spatiotemporal distribution characteristics, spatial correlation characteristics, spatial interaction characteristics and spatial spillover effect of HDCT and its impact mechanism, hoping to scientifically grasp the basic driving force and spatiotemporal principles of HDCT of this area. Through this research, we aim to provide a reference for the realization of high-quality collaborative and innovative development of cultural tourism in the Yangtze River Delta urban agglomeration and other regions in the world.

## Index system construction and methods

### Overview of the study area

According to “the Yangtze River Delta urban agglomeration development plan” issued by the Central Committee of the Communist Party of China and the State Council in 2016, the Yangtze River Delta urban agglomeration covers 26 cities in Jiangsu, Zhejiang, Anhui and Shanghai, which includes Shanghai municipality directly under the central government (SH); Nanjing (NJ), Wuxi (WX), Changzhou (CZh), Suzhou (SZ), Nantong (NT), Yancheng (YC), Yangzhou (YZ), Zhenjiang (ZJ) and Taizhou (TZh) in Jiangsu Province; Hangzhou (HZh), Ningbo (NB), Jiaxing (JX), Huzhou (HZ), Shaoxing (SX), Jinhua (JH), Zhoushan (ZS) and Taizhou (TZ) in Zhejiang Province; Hefei (HF), Wuhu (WH), Maanshan (MAS), Tongling (TL), Anqing (AQ), Chuzhou (CZ), Chizhou (ChZ) and Xuancheng (XC) in Anhui Province.

There are two reasons to choose the Yangtze River Delta Urban Agglomeration as a case study. On the one hand, it is a typical and representative region for studying HDCT. The region accounting for only 2.2% of the total area of country has always been a well-known tourist destination loved by tourists [31, 35]. In 2019, the region’s total revenue of tourism industry accounts for more than 40% of the whole country, and the foreign exchange income of tourism accounts for nearly 20% of the whole country. As the largest urban agglomeration in China and one of the six largest urban agglomerations in the world, with the most dynamic economy, the highest degree of openness and the strongest innovation ability, the region aims to become a world-class famous tourism urban agglomeration and a world-famous tourism destination. On the other hand, the region has outstanding geographical advantages, which is located in the center of East Asia and the key point of the East Asian route in the western Pacific Ocean. It is an important intersection zone between the "Belt and Road " and the Yangtze River economic belt. The region is rich in cultural resources, the number of World Heritage accounts for 14.5%, and national 5A scenic spots account for more than 20% of the country respectively, such as Mount Huangshan, Ancient villages in southern Anhui (Xidi and Hongcun), West Lake Cultural Landscape of Hangzhou and Classical Gardens of Suzhou, etc.

### Index system construction and data sources

Cultural tourism industry has broader connotation, wider range and more complex composition, there has been no generally accepted evaluation index system so far. HDCT is not simply the good or bad of cultural tourism products, but a comprehensive concept which includes cultural tourism resources, facilities and environment as the basis, and can be reflected by cultural tourism economic quality and cultural tourism collaborative quality [2, 36, 37]. Referring to the existing research [10, 13, 18, 32, 38], this paper constructs a comprehensive evaluation index system with 40 indicators from six dimensions of high-quality resource, high-quality facilities, high-quality economy, high-quality environment, high-quality innovation, and high-quality integration of culture tourism (Table 1). Among them, high-quality resource and environment of culture tourism reflect the resource endowments and environmental conditions of urban cultural tourism destinations from the perspective of supply. High-quality of facilities is to examine the support and guarantee capacity of urban cultural tourism destinations from the perspective of demand [1, 13, 37]. high-quality economy and integration are to evaluate the market profitability and transformation ability of culture resources, respectively.

**Table 1:** The evaluation index system of HDCT.

Criteria	Indicators
High-quality resource (*H*_*1*_)	X_1_ Number of intangible cultural heritages at the provincial and national level; X_2_ Number of national key cultural relics protection units; X_3_ Number of famous historical and cultural cities, towns and villages; X_4_ Number of local folk festivals and special activities; X_5_ Number of national and provincial cultural industry demonstration bases; X_6_ Number of cultural centers (stations) and art performance venues; X_7_ Number of museum per 100 people; X_8_ Number of public library per 100 people; X_9_ Number of national scenic spots; X_10_ Number of above 4A-level tourist attractions
High-quality facilities (*H*_*2*_)	X_11_ Number of star-rated hotels; X_12_ Number of travel agencies; X_13_ Number of tourist centers; X_14_ Proportion of employees in tertiary industry(%); X_15_ Traffic mileage density (km/10000 people); X_16_ Number of Public toilets per 10,000 people; X_17_ Number of hospital beds per 10,000 people; X_18_ Number of buses per 10,000 people
High-quality economy (*H*_*3*_)	X_19_ Growth rate of total tourism revenue (%); X_20_ Growth rate of domestic tourism revenue (%); X_21_ Growth rate of domestic tourists (%); X_22_ Growth rate of international tourism receipts (%); X_23_ Growth rate of inbound tourists (%); X_24_ Proportion of total tourism revenue to GDP (%); X_25_ Proportion of total tourism revenue in added value of tertiary industry (%); X_26_ Growth rate of total tourism revenue (%); X_27_ Average stay days of tourists; X_28_ Ratio of the number of scenic spots to the total tourism revenue (%)
High-quality environment (*H*_*4*_)	X_29_ Days with good ambient air quality; X_30_ Green coverage (%); X_31_ Per capita green space area (m^2^); X_32_ Comprehensive utilization rate of general industrial solid waste (%); X_33_ Harmless treatment rate of domestic waste (%); X_34_ sewage treatment rate (%)
High-quality innovation (*H*_*5*_)	X_35_ Number of invention patents authorized per 10,000 people; X_36_ Number of college students per 10,000 people; X_37_ Per capita R&D expenditure; X_38_ Proportion of science expenditure in local financial expenditure (%)
High-quality integration (*H*_*6*_)	X_39_ Cultural tourism coupling degree (*C*); X_40_ Cultural tourism coordination development degree (*H*)

The six dimensions and their indicators are explained in detail as follows:

1. Resources are the foundation and core attraction of cultural tourism high-quality development. The criterion of high-quality resource (*H*_*1*_) mainly considered some indicators that could reflect the scale, abundance and taste of cultural tourism resources [39] and their transformation ability to products [13].
2. Facilities are the guarantee to meet the needs of tourists. The criterion of high-quality facilities (*H*_*2*_) mainly reflects the ability of cultural tourism industry to receive, support and guarantee the tourists’ activities [1, 19, 28, 40], including accommodation, transportation, staffing, etc.
3. Economy is the result of culture tourism high-quality development. The criterion of high-quality economy (*H*_*3*_) is mainly evaluated according to these indicators reflecting the market scale, revenue, contribution ability, and their growth rate of cultural tourism [19, 23, 31].
4. Environment is the prerequisite for culture tourism high-quality development because environment is not only the main activity place for tourists, but also an important part of culture tourism resources. The criterion of high-quality environment (*H*_*4*_) mainly focuses the natural external conditions of destinations in the narrow sense, and the government ability of environmental governance [18, 19, 26, 31].
5. Innovation is an important driving force for culture tourism high-quality development. The criterion of high-quality innovation (*H*_*5*_) mainly considers talent, science and technology, capital and other indicators [41–43].
6. Integration is the main tone of culture tourism high-quality development. The criterion of high-quality integration (*H*_*6*_) mainly focuses the integration level and coordination degree of culture and tourism [1, 2, 19].

The data of each indicator selected are from EPS (Easy Professional Superior) Database, specifically including China City Database, China Tourism Database, Chinese Culture Database, Chinese Regional Economic Database, Chinese Macroeconomic Database, and from China’s economic and social big data research platform of CNKI (China National Knowledge Infrastructure), mainly including Shanghai Statistical Yearbook, Zhejiang Statistical Yearbook, Jiangsu Statistical Yearbook, Anhui Statistical Yearbook, Chinese Culture and Related Industries Statistical Yearbook, Chinese Cultural Heritage Statistical Yearbook. Some missing data can be supplemented by consulting the statistical bulletin of national economic and social development published on the internet and the official website of culture and tourism department. In 2011, Anhui provincial government carried out zoning adjustment by changing Chaohu from a prefecture-level city to a county-level city, which was finally managed by Hefei city. Therefore, the data of Chaohu, which was separately included in the statistics before 2011, was added to Hefei city according to the latest division of urban units.

### Methods

#### Quality evaluation

The linear weighting method is suitable for measuring the comprehensive level of each index which is relatively independent. Therefore, this method is used for quantitative measurement of the high-quality development level of culture tourism in the Yangtze River Delta urban agglomeration. The calculation method refers to the research of two scholars [44, 45]. The specific formula is as follows:

$$u_{i}=\sum_{i=1}^{n}w_{ij}\times u_{ij}$$ (1)

Where, U_ij_ is the quality level of j city in the i year, and reflects the efficacy contribution of index j to the system, W_ij_ is the index weight. The efficacy contribution is calculated by the efficacy function according to the specific effect of each index on the system. Since the influence of each index in this paper is positive, the positive efficacy function should be used to quantify the original data:

$$u_{\mathrm{ij}}=(X_{ij}-X_{j\min})/(X_{j}\max-X_{j}\min)$$ (2)

Where, the value range of U_ij_ is 0 ~ 1, X_ij_ is the value of j index in the i year, and X_jmax_ and X_jmin_ are the maximum and minimum values of j index.

The weight of each index is calculated and measured by entropy method to avoid the subjectivity brought by experience weighting method and solve the problem of information overlap among multiple index variables [28, 30]. The coupling coordination degree model is used to construct the indicator of cultural tourism coupling degree (*C*), whose formula is as follows [44–46]:

$$D=\sqrt{CT},\;T=\varphi U_{1}+\lambda U_{2}$$ (3)

$$C=2\sqrt{U_{1}+U_{2}}/\left[U_{1}+U_{2}\right],U_{1}=\sum a_{m}X_{m}^{\prime },\;U_{2}=\sum b_{n}X_{n}^{\prime }$$ (4)

where *U*_1_ and *U*_2_ refer to the comprehensive level of culture and tourism industry, respectively. *X’m* and *X’n* represent the standardized values of the indicators of cultural and tourism industry, respectively. where *T* refers to the comprehensive coordination index of cultural and tourism industry. According to the existing study [5, 17, 31], both coefficient *φ* and *λ* are set to be 0.5.

Furthermore, the coordinated development degree matrix of the system of HDCT is established to measure the coordinated development relationship between each two criteria of HDCT. The formula is as follows:

$$H_{de}=\left|Q_{d}-Q_{e}\right|/\max(Q_{d},\;Q_{e})$$ (5)

where *H*_*de*_ represents the coordinated development degree between the *d*-th criterion and the *e*-th criterion of HDCT. *Q*_*d*_ and *Q*_*e*_ represents the comprehensive score of the *d*-th and *e*-th criterion of HDCT, respectively.

#### Spatiotemporal path analysis

Firstly, spatial autocorrelation is used to analyze the spatial aggregation characteristics and patterns of HDCT in Yangtze River Delta urban agglomeration including global spatial autocorrelation and local spatial autocorrelation [47], using global and local Moran’s *I* to evaluate the global and local spatial relationship of HDCT [47].

Secondly, in order to reveal the dynamic continuity of the above-mentioned static local spatial relations in time [48], LISA time path analysis is used to further investigate the spatiotemporal collaborative changes and dynamic characteristics of HDCT which includes relative length (*Γ*_*i*_) and bending degree (*ζ*_*i*_). The formula is as follows [47, 49]:

$$\Gamma_{i}=n\sum_{t=1}^{T-1}d(L_{i,t},L_{i,t+1})/\sum_{i=1}^{n}\sum_{t=1}^{T-1}d(L_{i,t},L_{i,t+1})$$ (6)

$$\zeta_{i}=\sum_{t=1}^{T-1}d(L_{i,t},L_{i,t+1})/d(L_{i,1},L_{i,T}),\;L_{i,t}=z_{i,t}\sum_{j}w_{ij}z_{j,t}/\sum_{i}z_{i,t}^{2}$$ (7)

where *n* = 26. *T* is the length of time. *L*_*i*,*t*_ represents the position of the city *i* in the Moran’s *I* scatter plot in year *t*. *d*(*L*_*i*,*t*_,*L*_*i*,*t*+1_) represents the distance that city *i* moves from year *t* to year t+1; *z*_*i*,*t*_ is the development quality of z standardized cultural tourism in city *i* in year *t*, *w*_*ij*_ is the spatial weight matrix. *Γ*_*i*_ >1 means that the moving length of city *i* during the study period is larger than the mean value, otherwise *Γ*_*i*_ <1. The larger *Γ*_*i*_ is, the more dynamic local spatial dependence and spatial structure of the HDCT of city *i* have. If the moving path of the city *i* is not a straight line, then *ζ*_*i*_ >1, otherwise *ζ*_*i*_ <1. The larger the *ζ*_*i*_ is, the more curved the moving path is, the greater the effect of spatiotemporal dependence of the local structure of HDCT of city *i* is, and the more circuitous the local structure’s time variation is.

Finally, the spatiotemporal transition division method and transferring probability matrix are used to describe the evolution of local Moran’s *I* scatter plot among different local spatial types, in order to reveal the LISA spatial direction, change of HDCT [48, 49]. Spatiotemporal transition has four types: Type I represents the city itself and its neighbors are in stable condition, including LL_*t*_→LL_*t*+1_, LH_*t*_→LH_*t*+1_, HH_*t*_→HH_*t*+1_, HL_*t*_→HL_*t*+1_. Type II represents transition only occurs in the city itself, including LL_*t*_→HL_*t*+1_, LH_*t*_→HH_*t*+1_, HH_*t*_→LH _*t*+1_, HL_*t*_→LL_*t*+1_. Type III represents transition only occurs in neighboring cities, including LL_*t*_→LH_*t*+1_, LH_*t*_→LL_*t*+1_, HH_*t*_→HL_*t*+1_, HL_*t*_→HH_*t*+1_. Type IV represents transition occurs in the city itself and its neighboring cities, including LL_*t*_→HH_*t*+1_, LH_*t*_→HL_*t*+1_, HH_*t*_→LL_*t*+1_, HL_*t*_→LH _*t*+1_.

#### Spatial Durbin Model (SDM)

HDCT of a city is a kind of spatial statistical value containing location information, which is influenced by its neighboring cities, and has spatial spillover and interactive effects. Considering the spatial correlation of geographical elements, spatial econometric model with spatial weight matrix can better reflect the spatial effect and their influencing factors [48]. Spatial Durbin Model (SDM) includes both endogenous and exogenous interaction effects with a more general form, and can effectively estimate the impact effect of HDCT, thus, SDM is chosen to estimate the impact mechanism of CTDQ. The formula is as follows [50, 51]:

$$Y_{i,t}=\delta \sum_{j=1}^{n}w_{ij}Y_{j,t}+\alpha X_{i,t}+\beta \sum_{j=1}^{n}w_{ij}X_{j,t}+\mu_{i}+\nu_{t}+\varepsilon_{i,t}$$ (8)

where *Y*_*i*,*t*_ and *X*_*i*,*t*_ are the HDCT and its influencing factors of city *i* in year *t*. *δ* is the coefficient of spatial lag term. *μ*_*i*_, *ν*_*t*_, and *ε*_*i*,*t*_ represent individual effect, temporal effect, and error term, respectively. *α*, *β* are the estimated coefficient and spatial interaction coefficient of influencing factors. If *β* = 0, *δ* ≠ 0, formula (8) is SLM; if *β+αδ* = 0, formula (8) is SEM [51].

## Results

### Spatiotemporal distribution characteristics of HDCT

**Fig 1:**
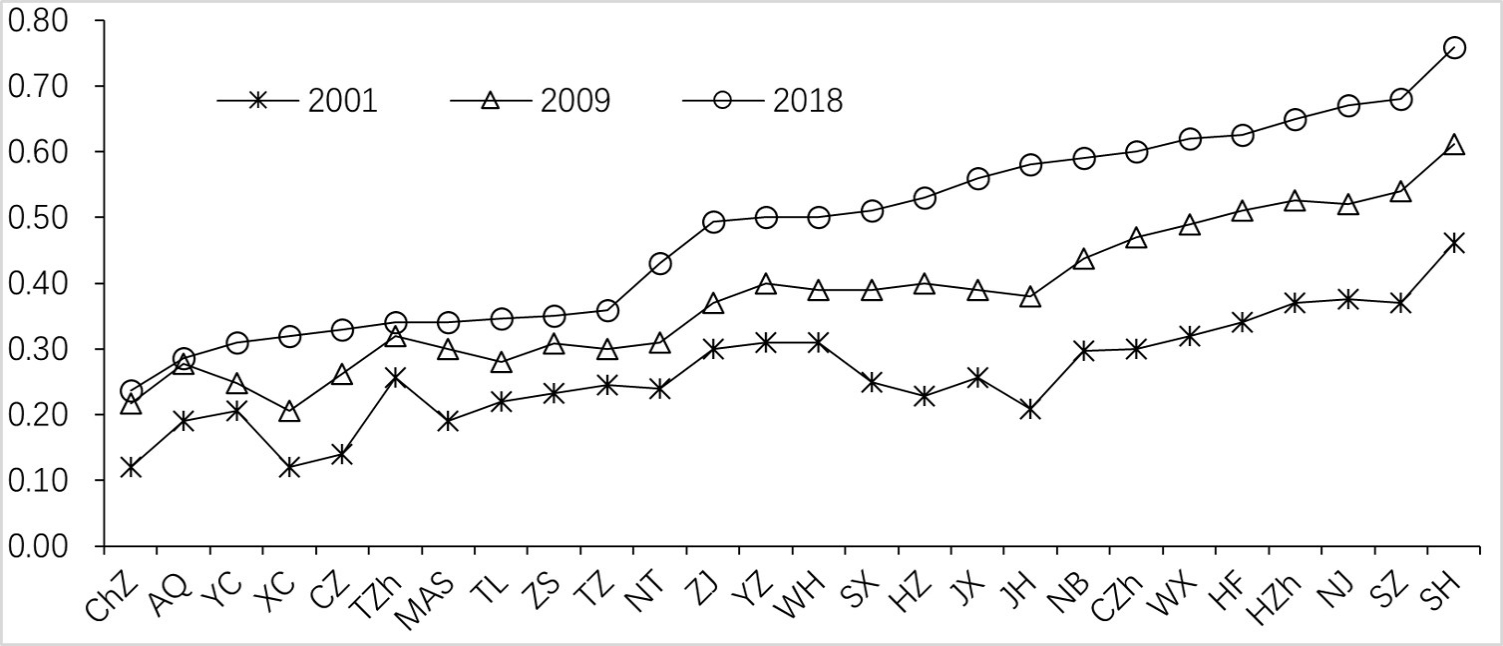
Level of HDCT of 26 cities.

**Fig 2:**
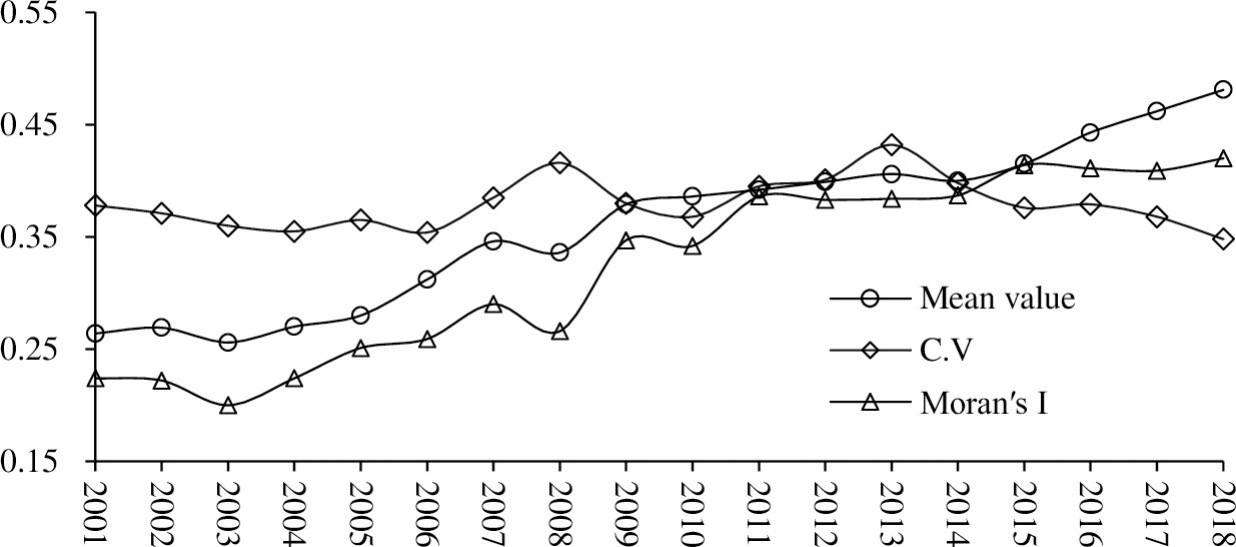
The mean value and C.V of HDCT and its global Moran’s I.

During the study period, HDCT of 26 cities in the Yangtze River Delta urban agglomeration shows a fluctuating upward trend, 21 cities accounting for 80.77% are generally at a medium and high level in 2018 (Fig 1). The HDCT has been improved in varying degrees, specifically, the lowest and highest level of HDCT increased from 0.12 and 0.461 in 2001 to 0.237 and 0.759 in 2018 (Fig 2), and the average increased from 0.264 in 2001 to 0.481 in 2018. Shanghai has been always ranked first with an obvious advantage. Provincial capital cities of Nanjing, Hangzhou and Hefei, other cities such as Suzhou, Wuxi, Changzhou of Jiangsu Province, and Ningbo, Jinhua, Jiaxing, Huzhou of Zhejiang Province are in the top 10, which these cities have a large growth rate with a strong stability of position.

There are some differences in the level of HDCT among 26 cities, but the difference is decreasing year by year (Fig 2). The coefficient of variation (C.V) of HDCT in different cities ranged from 0.348 to 0.432 from 2001 to 2018, except for the peaks of 0.416 and 0.432 in 2008 and 2013, it has been slightly decreased after 2008. The overall non-equilibrium of HDCT in the Yangtze River Delta urban agglomeration is relatively weak, and its overall fluctuation range is comparatively small, indicating that the difference of the HDCT in this region has gradually narrowed.

Considering that the HDCT has not yet formed a unified classification standard [25, 27], we use Jenks natural breaks method to divide the level of HDCT into four types: HDCT>0.460 means high level, 0.339<HDCT≤0.460 means medium and high level, 0.239<HDCT≤0.339 means medium and low level, HDCT≤ 0.239 means low level. As a default data classification method in ArcGIS mapping, Jenks natural breaks method are widely used in spatial data classification, whose classification principle is that the inter group variance is as large as possible, and the intra group variance is as small as possible.

As shown in Table 2, the number of cities with high level of HDCT has increased significantly, and the spatial agglomeration degree continues to strengthen with an obvious Z-shaped characteristic. 15 cities accounting for 57.69% have entered the stage of high-quality tourism development in 2018, compared with only 1 in 2001 and 7 in 2009. In addition to Shanghai, other 14 high level cities of HDCT mainly included six in Jiangsu Province, six in Zhejiang Province and two in Anhui Province. Chizhou, Anqing, Chouzhou and Xuancheng of Anhui Province have been in a low-level state and slow growth which the development lag disadvantage is comparatively obvious.

**Table 2:** Type classification of HDCT.

Type	low level	medium and low level	medium and high level	high level
2001	XC, ChZ, CZ MAS, AQ, YC, JH TL, HZ, ZS	NT, TZ, SX, JX, TZh, NB ZJ, CZh, WH, YZ, WX	HF, HZh, SZ, NJ	SH
2009	XC, ChZ	YC, CZ, AQ, TL, MAS	ZJ, JH, SX, WH	CZh, WX, HF, NJ
		TZ, ZS, NT, TZh	JX, HZ, YZ, NB	HZh, SZ, SH
2018	ChZ	AQ, YC, XC, CZ	MAS, TZh, TL	ZJ, WH, YZ, SX, HZ
				JX, JH, NB, CZh, WX
			ZS, TZ, NT	
				HF, HZh, NJ, SZ, SH

### Spatiotemporal association characteristics of HDCT

As shown in Fig 2, global Moran’s *I* of HDCT during 18 years were positive, rising from 0.213 in 2001 to 0.437 in 2018, and all passed the significance test of 0.05, indicating that HDCT of different city has a strong positive spatial autocorrelation, and the spatial proximity of similar cities is significant, that is, the cities with high level of HDCT tend to be adjacent in space, and the cities with low level of HDCT tend to be adjacent in space too. The spatial effect cannot be ignored when investigating the spatial characteristics of HDCT and its impact mechanism.

Using local spatial autocorrelation to further explore the evolution characteristics of local spatial correlation, the LISA clustering results could be divided into five clustering types of not significant (No), high-high (HH), high-low (HL), low-high (LH) and low-low (LL) types. Except for the not significant cities, the high-high and low-low type cities of HDCT tended to be more and more clear in space. The local pattern of spatial association of HDCT is mainly dominated by high-high and low-low agglomeration (Table 3), which gradually highlights the polarization characteristics of positive spatial correlation in southwest Anhui and Shanghai metropolitan area.

**Table 3:** The LISA clustering of HDCT.

Type	LL	LH	HL	HH
2001	TL	HZ	HZh	SH, SZ, WX
2009	TL, ChZ	ZS	CZh	SH, SZ, WX, HZ
2018	TL, AQ, ChZ	NT	HZh, NJ	SH, SZ, WX, HZ, CZh

The cities of high-high concentration are relatively stable in Shanghai, Suzhou, and Wuxi. After 2009, Huzhou and Changzhou have been successively increased. High quality tourism cities gradually show obvious spatial spillover and radiation effect on adjacent cities. The cities of low-low concentration are mainly distributed in southwest Anhui, such as Tongling, Anqing and Chizhou. Cities of high-low and low-high concentration were relatively small, and mainly located in Nantong and Zhoushan after 2009.

### Spatiotemporal path of HDCT

With the help of the relative length, curvature and transition direction of LISA time path, this paper analyzed the dynamics of the local spatial structure, the volatility of spatial dependence direction and spatial integration of HDCT (Table 4). The results show the spatial distribution of HDCT has strongly spatial stability, but the time sensitivity in the direction of spatial dependence is weak and lack of spatial integration.

**Table 4:** LISA time path evolution of HDCT.

relative length	City	curvature	City	direction	City
0.0213~0.1569	WH, NJ, TL, CZh, YC, WX, YZ	1.0001~1.0875	HZ, YZ, HZh, WH, TZ, TZh, NB, AQ, JH, SX, ZS, TL, SH, XC	0°~90°	WX, SZ, JX, CZh
					HZ, NB, JH, SX
0.1570~0.2792	CZ, SX, ZJ, HZ	1.0876~1.3407	WX, NJ, SZ, YC, CZ, JX, CZh	90°~180°	NT, ZJ, NJ, HZh, WH, TZ, ZS, TL, SH
0.2793~0.6297	NB, HF, SZ, NT, ChZ, MAS, SH, ZS, TZ	1.3408~3.0027	HF, ZJ, ChZ	180°~270°	YC, TZh, AQ
0.6298~1.1247	HZh, JX, AQ, XC, TZh, JH	3.0028~5.1346	NT, MAS	270°~360°	MAS, HF, ChZ, CZ YZ, XC

(1) In terms of relative length, Jinhua is the only city with a relative length greater than 1, indicating that the stability of spatial pattern of HDCT is comparatively strong, and the path dependence effect is obvious. The cities with large relative length included Taizhou, Xuancheng, Anqing, and Jiaxing, which were slow in the early stage, but fast in the later stage in cultural tourism development, thus showing obvious dynamic changes. Wuhu is the city with the shortest relative length (0.021). (2) In terms of curvature, the change is not obvious. There are 14 cities in the lower level, accounting for 53.8%, which indicates that the fluctuation of each city and its adjacent cities in the direction of spatial dependence is relatively stable. Maanshan had the largest curvature (5.135), followed by Nantong (3.011), and Hefei (2.248), reflecting that these cities and their neighboring cities are significantly affected by spatiotemporal dependence, and the neighboring cities have a greater impact on them. (3) In terms of direction, there are 11 cities with coordinated growth (0°~90°, 180°~270°), accounting for 42.3%, showing that the spatial integration of HDCT’s evolution is insufficient. Specifically, the transition directions of 8 cities including Wuxi, Suzhou, Jiaxing, Changzhou, Huzhou, Ningbo, Jinhua, Shaoxing and their neighboring cities were positively coordinated growth. The transition directions of 3 cities including Anqing, Yancheng, Taizhou and their neighboring cities were negatively coordinated movement, while the other 15 cities had the opposite transition direction to their neighboring cities.

The spatiotemporal transition method proposed by Rey et al. (2011) is used to analyze the transfer of the Local Moran’s *I* scatter diagram in local spatial association types, in order to reveal the LISA time path moving characteristics and transfer probability of HDCT in the Yangtze River Delta urban agglomeration (Table 5).

**Table 5:** Transition probability matrices of local Moran′s I.

*t*/*t*+1	HH	LH	LL	HL	Types	*n*	Proportion
HH	0.83	0.17	0.00	0.00	I	480	68.87%
LH	0.33	0.50	0.17	0.00	II	75	10.76%
LL	0.00	0.00	1.00	0.00	III	46	6.60%
HL	0.60	0.20	0.00	0.20	IV	17	1.58%

**Fig 3:**
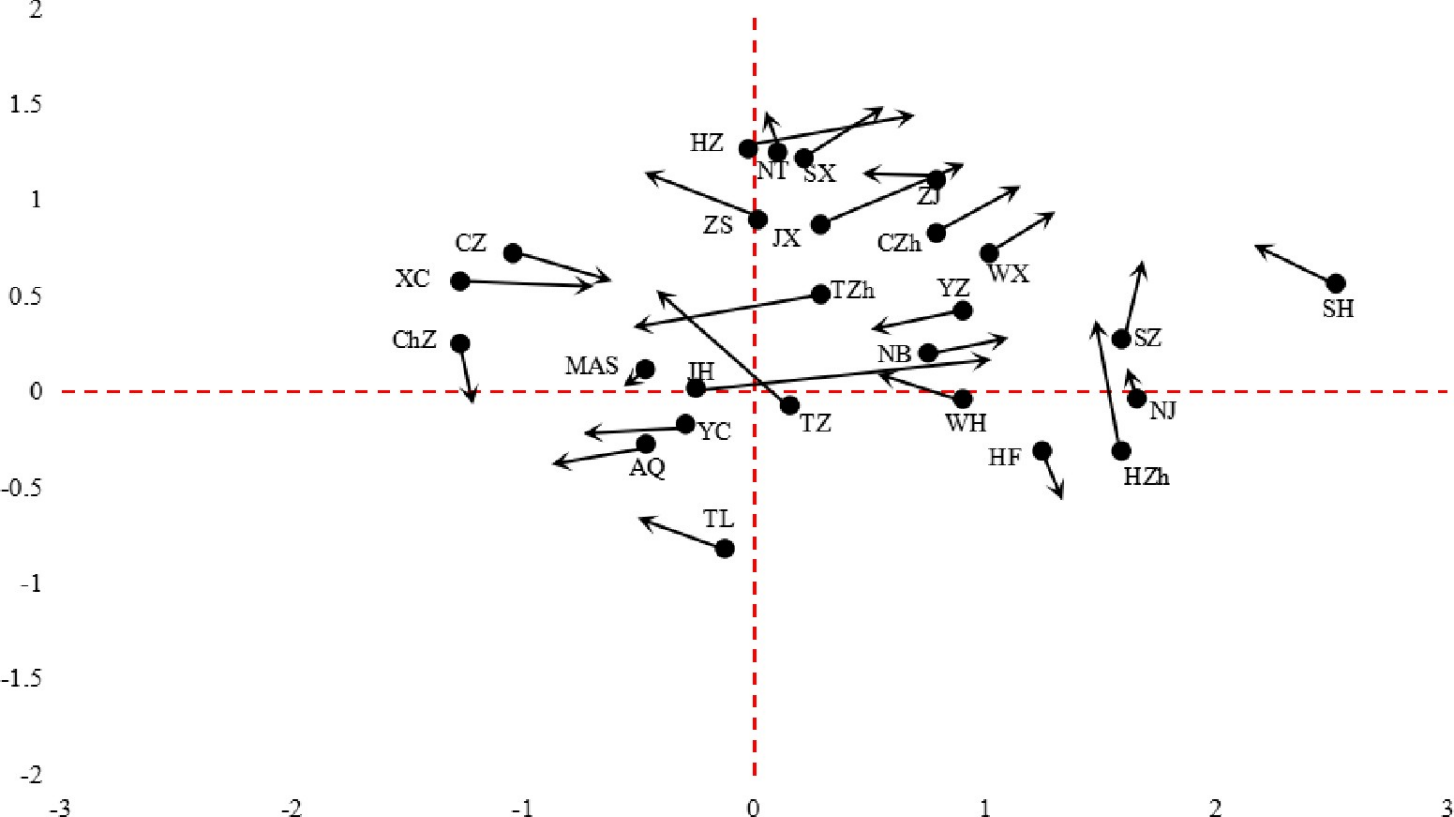
LISA time path movement feature of HDCT. Cities of HH type include NT, SX, ZJ, **ZS**, JX, CZh, SH, WX, **TZh**, YZ, SZ, and NB. Cities of LH type include **HZ**, CZ, XC, MAS, **ChZ**, and **JH**. Cities of LL type include TL, YC, and AQ. Cities of HL type include **NJ**, **HZh**, HF, **WH**, and **TZ**, and cities’ abbreviations in bold refers to these cities’ HDCT undergone time-space transitions.

The most common transition is Type I, accounting for 68.87%, that is, no transition has occurred in local city and its neighboring cities. From 2001 to 2018, there were 132 type transitions, of which the ratios of type II, type III, and type IV was 10.76%, 6.6%, and 1.58%, respectively. The spatiotemporal transition mainly includes 5 types of HH_*t*_→LH_*t*+1_, LH_*t*_→HH _*t*+1_, LH_*t*_→LL _*t*+1_, HL_*t*_→HH_*t*+1_, HL_*t*_→LH_*t*+1_. Among them, the transfer probability (0.6) of HL_*t*_→HH_*t*+1_ is the largest, followed by LH_*t*_→HH _*t*+1_ (0.33), and the transfer probabilities of other types are lower, which shows that the high value Club agglomeration characteristics of HDCT is enhanced, the strong cumulative effect of the central city and the regional integration development will effectively boost the tourism quality of surrounding cities, and the overall space is significantly improved through the spillover effect. As can be seen from Fig 3, only 9 cities have experienced the transition between different quadrants during the study period, which indicates that the spatiotemporal evolution of HDCT had certain lock-in effect and path dependence, and the switching between different types has inert.

## Impact mechanism of HDCT

### Factors selection

The spatiotemporal evolution of HDCT are complicated processes under the interaction of multiple factors. From 6 aspects of economic development level, advanced industrial structure, economic outreach, technological innovation, transportation level and professional talents [28, 30, 50, 52], we choose per capita GDP (*Pgdp*), ratio of output value of tertiary industry to secondary industry (*Str*), actual use of foreign direct investment (*FDI*), the proportion of science and technology expenditure in the general budget expenditure of local finance in the GDP (*Tec*), density of traffic network (*Tra*), and the number of university students per 10,000 (*Tal*) as the determinants to explore their impact on HDCT.

In order to determine the final form of the formula (8), the (Robust) LM test is used to compare SEM and SLM. LM-lag, Robust LM-lag, LM-error and Robust LM-error all passed the significance test of 0.01, showing that spatial dependence of the interpretation model of HDCT has both spatial lag term and error term. Thus, we establish SDM and determine whether SDM can be simplified to SEM or SLM through the Walds and LR test [50]. The test results of Wald-spatial error, Wald-spatial lag, LR-spatial error, and LR-spatial lag also passed 0.01 significance test, which means that SDM of HDCT cannot be simplified to SLM or SEM. In the end, the Hausman test results reject random effects and accept SDM with fixed effects (Table 6). In the SDM regression results of HDCT, the *Log L* and *Adj*.*R*^*2*^ values of spatial fixed effect (SF) are bigger than those without fixed effect (NF), time fixed effect (TF), and space-time fixed effect (STF), indicating that the estimation results of SF should be selected for regression parameter interpretation.

**Table 6:** SDM results for HDCT.

variables	NF	TF	SF	STF	variables	NF	TF	SF	STF
*Pgdp*	0.231***	0.358***	0.367***	0.249**	W* *Pgdp*	-0.182*	0.236**	0.251***	0.281*
*Str*	-0.276*	0.271***	0.361*	-0.291**	W* *Str*	0.435***	0.246***	-0.334**	0.382*
*FDI*	0.125*	0.324*	0.144**	0.193	W* *FDI*	0.118*	0.209*	0.191	0.121
*Tec*	0.276**	0.134**	0.379***	0.343*	W* *Tec*	0.277**	0.246*	0.272***	0.283*
*Tra*	0.181*	0.201**	0.231*	0.215*	W* *Tra*	-0.029	-0.186*	-0.089**	-0.115**
*Tal*	0.172	0.092**	0.191*	0.289	W*Tal	0.047*	0.109	0.113*	0.179**
*Adj*.*R*^*2*^	0.828	0.786	0.886	0.499	*δ*	0.113*	0.319*	0.256**	-0.077
*Log L*	-173.8	-158.3	-84.61	-89.36					

Note:

***, **, and * indicates significance at the 0.01, 0.05, and 0.10 level, respectively. The same below.

### Impacting effect

As shown in Table 6, the spatial spillover coefficient *δ* of HDCT is 0.256, which has passed the significance test of 0.05, indicating that there is a significant spatial spillover effect in the HDCT in the Yangtze River Delta urban agglomeration, and the direct and spatial interaction effects of various factors are obvious. The elasticity coefficients of each factor on HDCT is significantly positive, which indicates that the level of economic development, advanced industrial structure, the degree of economic outreach, technological innovation, transportation level, and professional personnel are important means to directly improve HDCT. The elastic coefficients of *Pgdp*, *Tec*, and *Tal* with spatial lag are significantly positive, while those of *Str* and *Tra* are significantly negative, indicating that economy, technology, and talents have significant spatial spillover effects on HDCT. Strengthening their regional flow will help to promote the HDCT of different city. However, the advanced industrial structure and the improvement of regional traffic will restrain the HDCT in neighboring cities through the effect of spatial competition.

The total effect of the influencing factors of HDCT from large to small is the economic development level > technological innovation > professional talents > advanced industrial structure > transportation level > economic outreach. By decomposing the estimated coefficients (Table 7) into direct and indirect effects, the marginal effects of various factors on HDCT are further examined. As shown in Table 4, the direct effect coefficients of each factor from large to small are *Pgdp*, *Str*, *Tec*, *Tal*, *Tra* and *FDI*. Each positive change of these factors will promote HDCT by 0.323, 0.312, 0.267, 0.197, 0.183, and 0.165. At present, economic development is still the most important antecedent for high-quality development of cultural tourism [7, 19, 23]. The optimization of industrial structure and technological innovation will promote the transformation of cultural tourism industry from labor and capital-intensive to technology and knowledge-intensive, and ultimately improve the HDCT. The indirect effect coefficients of each factor from large to small are *Tec*, *Pgdp*, *Tal*, *Tra*, and *Str*. Each positive change of the first three factors will promote HDCT in neighboring cities by 0.211, 0.206, 0.072, and each positive change of the last two factors will cause HDCT in neighboring cities to decrease by 0.048 and 0.108, respectively. The indirect effect coefficient of *FDI* is not significant, indicating that whether the increase of foreign investment has an impact on HDCT in neighboring cities has not yet been tested.

**Table 7:** Decomposition results of spatial effect.

variables	Pgdp	Str	FDI	Tec	Tra	Tal
Direct effect	0.323***	0.312***	0.165**	0.267**	0.183**	0.197**
Indirect effect	0.206***	-0.108**	-0.116	0.211**	-0.048*	0.072*
Total effect	0.529***	0.204*	0.049	0.478**	0.135*	0.269**

### Division of evolution type

In order to further depict the internal mechanism of the formation of HDCT’s spatiotemporal path, the impact factors with cities of different spatiotemporal transition types are used as explanatory variables into the multiple linear regression model for parameter estimation, and four evolution types are classified according to the dominant factors in the estimated results (Table 8). 17 cities of Type I representing the stability of the city itself and its neighboring cities is the largest, such as Nantong, Shaoxing, and so on, which are mainly affected by economic development level (0.478). Therefore, this type of cities is named as economic stabilizing type. 4 cities (Huzhou, Jinhua, Zhoushan, and Taizhou) of type II with their own transition are most obviously affected by advanced industrial structure (0.367), so it is named as industry optimizing type. 4 cities (Nanjing, Hangzhou, Wuhu, and Chizhou) of type III only with their neighboring cities’ transition are mainly driven by technological innovation (0.343) and named as innovation driving type. Taizhou (TZ) is the only type IV city representing the transition of itself and its neighboring cities, which is significantly affected by location transportation (0.278) and named as traffic impacting type.

**Table 8:** Evolution types of HDCT.

Evolution Types	Transition types	top three factors	Cities
Economy stabilizing type	LL_*t*_→LL_*t*+1_	*Pgdp*(0.478) *、Tal*(0.239)、 *Str*(0.239)	NT, SX, ZJ, JX, CZh, SH WX, YZ, SZ, NB, CZ, XC MAS, TL, YC, AQ, HF
	LH_*t*_→LH_*t*+1_		
	HH_*t*_→HH_*t*+1_		
	HL_*t*_→HL_*t*+1_		
Industry optimizing type	LH_*t*_→HH_*t*+1_	*Str*(0.367)、*Pgdp*(0.325) *、Tal*(0.186)	HZ, JH, ZS, TZh
	HH_*t*_→LH_*t*+1_		
Innovation driving type	HL_*t*_→HH_*t*+1_	*Tec*(0.343)、 *Str*(0.284) *、Pgdp*(0.273)	NJ, HZh, WH, ChZ
	LH_*t*_→LL_*t*+1_		
Traffic impacting type	HL_*t*_→LH_*t*+1_	*Tra*(0.278)、 *Tec*(0.261)、 *Pgdp*(0.214)	TZ

## Discussion

The high-quality development of cultural tourism in Yangtze River Delta urban agglomeration is an important support for the region to build a world-class and high-quality tourism destination. Under the strategic background and realistic demand of the integrated development of culture tourism industry, selecting this typical area as an example to explore the spatiotemporal evolution of HDCT and its impact mechanism has certain theoretical contribution and practical reference value to realize high quality collaborative innovation development of regional cultural tourism [9].

There are two methods of single index and multi-index to evaluate industrial development quality. The single index method has the characteristics of comparability and operability, but is not easy to reflect the comprehensive situation of industrial development [53], multi-index method is more meaningful for scientific evaluation. According to this research, the HDCT of most cities in Yangtze River Delta urban agglomeration is at a medium and high level, which indicates that urban tourism development pays more and more attention to the improvement of quality in the process of pursuing the quantity growth, the pace of high-quality development is obviously accelerated, the characteristics of spatial aggregation are gradually enhanced, and there are significant spatial spillover effects among them.

However, the level of HDCT in cities located in Southwest Anhui, Southwest Zhejiang and Northern Jiangsu is relatively low, the improvement speed is slow, and the regional spatial imbalance is still serious. The finding of local spatial association pattern with high-high and low-low types further confirms that the imbalanced development exists not only in China’s economy, but also in its cultural tourism industry [9, 30]. Each city should put forward differentiated and targeted policies to improve the overall quality of cultural tourism development according to its own reality and development stage.

The path dependence and lock-in effect of local correlation structure of HDCT show that the spatial distribution of HDCT is stable, the aggregation characteristics are gradually obvious, and the transfer between types has a certain inertia. Through the analysis of impact factors, it is quantitatively confirmed that there is a significantly spatial spillover effect of HDCT among 26 cities. The strong accumulation effect of central city will effectively promote the improvement of HDCT in neighboring cities through spatial spillover effect. Compared with previous studies [13, 32], this is a new discovery that highlights the location information of spatial element, which can help to deepen the understanding of the relationship of HDCT among cities and provide some reference for future work, such as the Pearl River Delta, Beijing-Tianjin-Hebei urban agglomeration.

The spatiotemporal evolution of HDCT is the result driven by many determinants, rather than mainly influenced by a single factor [6, 11, 52–53]. Although it is subjective to divide the evolution types of cities’ HDCT into four types according to the dominant factor, it can still reflect the regional characteristics of the sustainable development ability of culture tourism in Yangtze River Delta urban agglomeration to a certain extent. The development of tourism industry traditionally relies on natural resources and low-level labor input, continuous capital investment, and development construction [54], which is also the main driving force for the high-quality development of urban cultural tourism, accounting for 65.4%. The change of growth mode is the inevitable requirement of sustainable development of tourism [55]. With the proposal of the strategy of "invigorating tourism with science and technology", scientific and technological innovation has gradually become the engine of the high-quality development of tourism. Some cities such as Hangzhou and Nanjing have achieved certain results, and we hope these findings could provide reference for the policy-makers.

Furthermore, the paper also has several limitations. On the one hand, in view of the comprehensiveness and complexity of the concept and connotation of culture tourism, the selection of indicators still needs to be improved. The high-quality development of culture tourism covers multiple fields and some input and output data are hard to collect and quantify. For example, tourism is an important reference to measure the living standards of the people [56]. The high-quality development of cultural tourism is closely related to the sense of acquisition and recognition of residents and tourists. The evaluation results will be more accurate and valuable if the residents’ sense of acquisition and tourists’ well-being are included. On the other hand, in the part of impact mechanism analysis, we need to build a more comprehensive influencing factors system (such as tourist satisfaction data), and further explore the heterogeneity of spatial spillover effect, which will enrich the research results.

Cultural tourism has a more significant advantage in generating foreign exchange income and creating employment [39, 57]. The high-quality development of cultural tourism in Yangtze River Delta urban agglomeration is an important component to promote the implementation of the national strategy of the Yangtze River Delta integration and an important support to achieve the goal of building a world-famous tourist destination [2, 7, 13]. This paper objectively evaluated the level of HDCT in this region and analyzed its spatiotemporal evolution characteristics and impact mechanism from the perspective of quantitative research, which is only a preliminary exploration in the field of cultural tourism development quality research, highlighting the value of data and importance of space. Through this study, we aim to provide some reference for future research and the determination of related directions, so as to attract more scholars to pay more attention to the high-quality development of cultural tourism industry and other relevant issues. considering neighboring provinces (such as Shandong, Henan) may have spillover effect on our research area, in order to minimize this impact, it will better to choose nation-wide sample to study the HDCT. In addition, the comparative analysis with the Pearl River Delta and Beijing-Tianjin-Hebei urban agglomeration is more practical and meaningful.

## Conclusions

The main conclusions are as follows:

1. During the study period, the HDCT of 26 cities in the Yangtze River Delta urban agglomeration shows a fluctuating upward trend with a relatively weak regional imbalance, accounting for more than 80% of the cities at a medium and high level. By 2018, a total of 15 cities have entered the stage of high-quality tourism development, accounting for 57.69%, and showing a “Z” pattern in space. Shanghai has been always in the first place with obvious advantages, followed by Zhejiang province and Jiangsu province, and Anhui province is relatively backward.
2. The spatial autocorrelation of HDCT in all cities is significantly positive with the spatial clustering and proximity of the same kind increasing. The cities with high-level HDCT tend to be adjacent in space, and so do the low-level cities. The increasing number of high-quality tourism development cities shows urban tourism development has obviously spatial spillover and radiation effects. The local spatial association pattern was mainly dominated by HH and LL agglomeration. The polarization characteristic of positive spatial association is gradually prominent in Southwest Anhui and Shanghai metropolitan.
3. The relative length of LISA time path of HDCT is high in the south and low in the north, and the curvature is generally small, which indicates that the local spatial correlation structure has strong stability, obvious path dependence and spatial lock-in effect, and the switching between different types has certain transfer inertia. There are only 8 cities with positive synergetic growth, and the spatial integration of urban tourism development quality evolution is insufficient.
4. The spatiotemporal evolution of HDCT shows a complex process under the interaction of multiple factors. The HDCT of different cities has a significant spatial spillover effect (0.256), and the direct and indirect effects are obvious. The level of economic development, technological innovation and professional talents are the three main influencing factors. According to the dominant factors affecting the evolution of HDCT, it could be divided into four types: economy stabilizing type, industry optimizing type, innovation driving type and traffic impacting type.

## Supporting information

S1 Fig Level of HDCT of 26 cities. (TIF)

S2 Fig The mean value and C.V of HDCT and its global Moran’s I. (TIF)

S3 Fig LISA time path movement feature of HDCT. (TIF)

S1 Table The evaluation index system of HDCT. (DOCX)

S2 Table Type classification of HDCT. (DOCX)

S3 Table The LISA clustering of HDCT. (DOCX)

S4 Table LISA time path evolution of HDCT. (DOCX)

S5 Table Transition probability matrices of local Moran′s I. (DOCX)

S6 Table SDM results for HDCT. (DOCX)

S7 Table Decomposition results of spatial effect. (DOCX)

S8 Table Evolution types of HDCT. (DOCX)

S1 File Data of Level of HDCT of 26 cities. (XLSX)

S2 File Data of the mean value and C.V of HDCT and its global Moran’s I. (XLSX)

S3 File Data of LISA time path movement feature of HDCT. (XLSX)
