# Supplementary figures and images for: Spatiotemporal characteristics and impact mechanism of high-quality development of cultural tourism in the Yangtze River Delta urban agglomeration
Source: PLoS One. 2021 Jun 22;16(6):e0252842. doi: 10.1371/journal.pone.0252842 (PMC8219149; doi:10.1371/journal.pone.0252842)

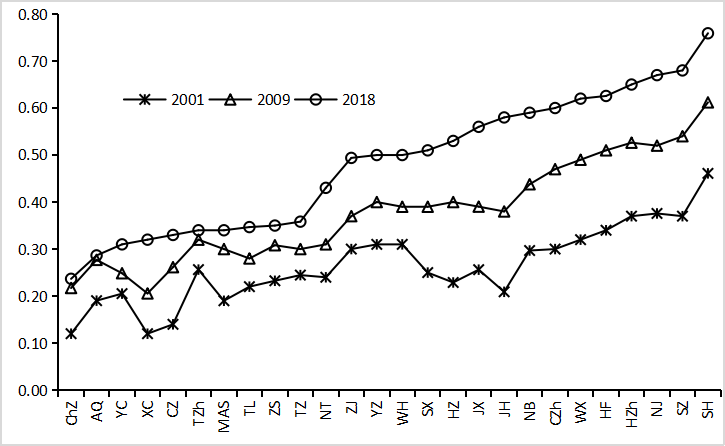

Supplement: S1 Fig — (TIF) [file pone.0252842.s001.tif]

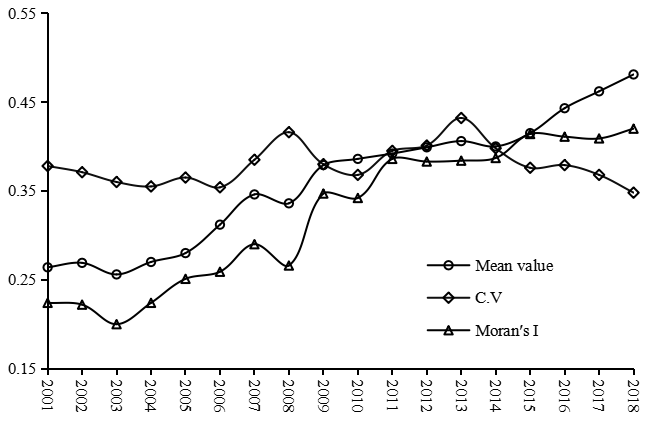

Supplement: S2 Fig — (TIF) [file pone.0252842.s002.tif]

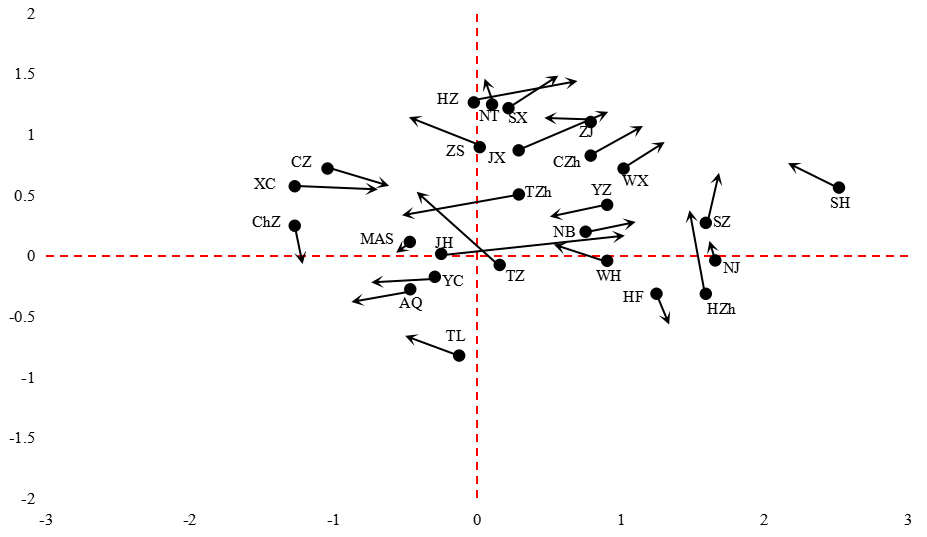

Supplement: S3 Fig — (TIF) [file pone.0252842.s003.tif]
