# Supplementary material for: Spatiotemporal characteristics and impact mechanism of high-quality development of cultural tourism in the Yangtze River Delta urban agglomeration
Source: PLoS One. 2021 Jun 22;16(6):e0252842. doi: 10.1371/journal.pone.0252842 (PMC8219149; doi:10.1371/journal.pone.0252842)
Supplement: S1 Table — (DOCX) [file pone.0252842.s004.docx]

**S1 Table. The evaluation index system of HDCT**

| Criteria | Indicators |
| --- | --- |
| High-quality resource (*H_1_*) | X_1_ Number of intangible cultural heritages at the provincial and national level; X_2_ Number of national key cultural relics protection units; X_3_ Number of famous historical and cultural cities, towns and villages; X_4_ Number of local folk festivals and special activities; X_5_ Number of national and provincial cultural industry demonstration bases; X_6_ Number of cultural centers (stations) and art performance venues; X_7_ Number of museum per 100 people; X_8_ Number of public library per 100 people; X_9_ Number of national scenic spots; X_10_ Number of above 4A-level tourist attractions |
| High-quality facilities (*H_2_*) | X_11_ Number of star-rated hotels; X_12_ Number of travel agencies; X_13_ Number of tourist centers; X_14_ Proportion of employees in tertiary industry(%); X_15_ Traffic mileage density (km/10000 people); X_16_ Number of Public toilets per 10,000 people; X_17_ Number of hospital beds per 10,000 people; X_18_ Number of buses per 10,000 people |
| High-quality economy (*H_3_*) | X_19_ Growth rate of total tourism revenue (%); X_20_ Growth rate of domestic tourism revenue (%); X_21_ Growth rate of domestic tourists (%); X_22_ Growth rate of international tourism receipts (%); X_23_ Growth rate of inbound tourists (%); X_24_ Proportion of total tourism revenue to GDP (%); X_25_ Proportion of total tourism revenue in added value of tertiary industry (%); X_26_ Growth rate of total tourism revenue (%); X_27_ Average stay days of tourists; X_28_ Ratio of the number of scenic spots to the total tourism revenue (%) |
| High-quality environment (*H_4_*) | X_29_ Days with good ambient air quality; X_30_ Green coverage (%); X_31_ Per capita green space area (m^2^); X_32_ Comprehensive utilization rate of general industrial solid waste (%); X_33_ Harmless treatment rate of domestic waste (%); X_34_ sewage treatment rate (%) |
| High-quality innovation (*H_5_*) | X_35_ Number of invention patents authorized per 10,000 people; X_36_ Number of college students per 10,000 people; X_37_ Per capita R&D expenditure; X_38_ Proportion of science expenditure in local financial expenditure (%) |
| High-quality integration (*H_6_*) | X_39_ Cultural tourism coupling degree (*C*); X_40_ Cultural tourism coordination development degree (*H*) |
