# Supplementary material for: Spatiotemporal characteristics and impact mechanism of high-quality development of cultural tourism in the Yangtze River Delta urban agglomeration
Source: PLoS One. 2021 Jun 22;16(6):e0252842. doi: 10.1371/journal.pone.0252842 (PMC8219149; doi:10.1371/journal.pone.0252842)
Supplement: S2 Table — (DOCX) [file pone.0252842.s005.docx]

| \| Type \| low level \| medium and low level \| medium and high level \| high level \| \| --- \| --- \| --- \| --- \| --- \| \| 2001 \| XC, ChZ, CZ MAS, AQ, YC, JH TL, HZ, ZS \| NT, TZ, SX, JX, TZh, NB ZJ, CZh, WH, YZ, WX \| HF, HZh, SZ, NJ \| SH \| \| 2009 \| XC, ChZ \| YC, CZ, AQ, TL, MAS  TZ, ZS, NT, TZh \| ZJ, JH, SX, WH  JX, HZ, YZ, NB \| CZh, WX, HF, NJ  HZh, SZ, SH \| \| 2018 \| ChZ \| AQ, YC, XC, CZ \| MAS, TZh, TL  ZS, TZ, NT \| ZJ, WH, YZ, SX, HZ  JX, JH, NB, CZh, WX  HF, HZh, NJ, SZ, SH \|   **S2 Table. Type classification of HDCT** |
| --- | --- | --- | --- | --- | --- | --- | --- | --- | --- | --- | --- | --- | --- | --- | --- | --- | --- | --- | --- | --- |
