# Supplementary material for: Spatiotemporal characteristics and impact mechanism of high-quality development of cultural tourism in the Yangtze River Delta urban agglomeration
Source: PLoS One. 2021 Jun 22;16(6):e0252842. doi: 10.1371/journal.pone.0252842 (PMC8219149; doi:10.1371/journal.pone.0252842)
Supplement: S3 Table — (DOCX) [file pone.0252842.s006.docx]

| \| Type \| LL \| LH \| HL \| HH \| \| --- \| --- \| --- \| --- \| --- \| \| 2001 \| TL \| HZ \| HZh \| SH, SZ, WX \| \| 2009 \| TL, ChZ \| ZS \| CZh \| SH, SZ, WX, HZ \| \| 2018 \| TL, AQ, ChZ \| NT \| HZh, NJ \| SH, SZ, WX, HZ, CZh \|   **S3 Table. The LISA clustering of HDCT** |
| --- | --- | --- | --- | --- | --- | --- | --- | --- | --- | --- | --- | --- | --- | --- | --- | --- | --- | --- | --- | --- |
