# Supplementary material for: Spatiotemporal characteristics and impact mechanism of high-quality development of cultural tourism in the Yangtze River Delta urban agglomeration
Source: PLoS One. 2021 Jun 22;16(6):e0252842. doi: 10.1371/journal.pone.0252842 (PMC8219149; doi:10.1371/journal.pone.0252842)
Supplement: S4 Table — (DOCX) [file pone.0252842.s007.docx]

| \| relative length \| City \| curvature \| City \| direction \| City \| \| --- \| --- \| --- \| --- \| --- \| --- \| \| 0.0213~0.1569 \| WH, NJ, TL, CZh, YC, WX, YZ \| 1.0001~1.0875 \| HZ, YZ, HZh, WH, TZ, TZh, NB, AQ, JH, SX, ZS, TL, SH, XC \| 0°~90° \| WX, SZ, JX, CZh  HZ, NB, JH, SX \| \| 0.1570~0.2792 \| CZ, SX, ZJ, HZ \| 1.0876~1.3407 \| WX, NJ, SZ, YC, CZ, JX, CZh \| 90°~180° \| NT, ZJ, NJ, HZh, WH, TZ, ZS, TL, SH \| \| 0.2793~0.6297 \| NB, HF, SZ, NT, ChZ, MAS, SH, ZS, TZ \| 1.3408~3.0027 \| HF, ZJ, ChZ \| 180°~270° \| YC, TZh, AQ \| \| 0.6298~1.1247 \| HZh, JX, AQ, XC, TZh, JH \| 3.0028~5.1346 \| NT, MAS \| 270°~360° \| MAS, HF, ChZ, CZ YZ, XC \|   **S4 Table. LISA time path evolution of HDCT** |
| --- | --- | --- | --- | --- | --- | --- | --- | --- | --- | --- | --- | --- | --- | --- | --- | --- | --- | --- | --- | --- | --- | --- | --- | --- | --- | --- | --- | --- | --- | --- |
