# Supplementary material for: Spatiotemporal characteristics and impact mechanism of high-quality development of cultural tourism in the Yangtze River Delta urban agglomeration
Source: PLoS One. 2021 Jun 22;16(6):e0252842. doi: 10.1371/journal.pone.0252842 (PMC8219149; doi:10.1371/journal.pone.0252842)
Supplement: S5 Table — (DOCX) [file pone.0252842.s008.docx]

**S5 Table. Transition probability matrices of Local Moran′s I**

| *t*/*t*+1 | HH | LH | LL | HL | Types | *n* | Proportion |
| --- | --- | --- | --- | --- | --- | --- | --- |
| HH | 0.83 | 0.17 | 0.00 | 0.00 | I | 480 | 68.87% |
| LH | 0.33 | 0.50 | 0.17 | 0.00 | II | 75 | 10.76% |
| LL | 0.00 | 0.00 | 1.00 | 0.00 | III | 46 | 6.60% |
| HL | 0.60 | 0.20 | 0.00 | 0.20 | IV | 17 | 1.58% |
