# Supplementary material for: Spatiotemporal characteristics and impact mechanism of high-quality development of cultural tourism in the Yangtze River Delta urban agglomeration
Source: PLoS One. 2021 Jun 22;16(6):e0252842. doi: 10.1371/journal.pone.0252842 (PMC8219149; doi:10.1371/journal.pone.0252842)
Supplement: S6 Table — (DOCX) [file pone.0252842.s009.docx]

**S6 Table. SDM results for HDCT**

| variables | **NF** | **TF** | **SF** | **STF** | variables | **NF** | **TF** | **SF** | **STF** |
| --- | --- | --- | --- | --- | --- | --- | --- | --- | --- |
| *Pgdp* | 0.231*** | 0.358*** | 0.367*** | 0.249** | W* *Pgdp* | -0.182* | 0.236** | 0.251*** | 0.281* |
| *Str* | -0.276* | 0.271*** | 0.361* | -0.291** | W* *Str* | 0.435*** | 0.246*** | -0.334** | 0.382* |
| *FDI* | 0.125* | 0.324* | 0.144** | 0.193 | W* *FDI* | 0.118* | 0.209* | 0.191 | 0.121 |
| *Tec* | 0.276** | 0.134** | 0.379*** | 0.343* | W* *Tec* | 0.277** | 0.246* | 0.272*** | 0.283* |
| *Tra* | 0.181* | 0.201** | 0.231* | 0.215* | W* *Tra* | -0.029 | -0.186* | -0.089** | -0.115** |
| *Tal* | 0.172 | 0.092** | 0.191* | 0.289 | W**Tal* | 0.047* | 0.109 | 0.113* | 0.179** |
| *Adj.R^2^* | 0.828 | 0.786 | 0.886 | 0.499 | *δ* | 0.113* | 0.319* | 0.256** | -0.077 |
| *Log L* | -173.8 | -158.3 | -84.61 | -89.36 |  |  |  |  |  |

Note: ***, **, and * indicates significance at the 0.01, 0.05, and 0.10 level, respectively. The same below.
