# Supplementary material for: Spatiotemporal characteristics and impact mechanism of high-quality development of cultural tourism in the Yangtze River Delta urban agglomeration
Source: PLoS One. 2021 Jun 22;16(6):e0252842. doi: 10.1371/journal.pone.0252842 (PMC8219149; doi:10.1371/journal.pone.0252842)
Supplement: S7 Table — (DOCX) [file pone.0252842.s010.docx]

**S7 Table. Decomposition results of spatial effect**

| variables | Pgdp | Str | FDI | Tec | Tra | Tal |
| --- | --- | --- | --- | --- | --- | --- |
| Direct effect | 0.323*** | 0.312*** | 0.165** | 0.267** | 0.183** | 0.197** |
| Indirect effect | 0.206*** | -0.108** | -0.116 | 0.211** | -0.048* | 0.072* |
| Total effect | 0.529*** | 0.204* | 0.049 | 0.478** | 0.135* | 0.269** |
