# Supplementary material for: Spatiotemporal characteristics and impact mechanism of high-quality development of cultural tourism in the Yangtze River Delta urban agglomeration
Source: PLoS One. 2021 Jun 22;16(6):e0252842. doi: 10.1371/journal.pone.0252842 (PMC8219149; doi:10.1371/journal.pone.0252842)
Supplement: S8 Table — (DOCX) [file pone.0252842.s011.docx]

**S8 Table. Evolution types of HDCT**

| Evolution Types | Transition types | top three factors | Cities |
| --- | --- | --- | --- |
| Economy stabilizing type | LL*_t_*→LL*_t_*_+1_  LH*_t_*→LH*_t_*_+1_  HH*_t_*→HH*_t_*_+1_  HL*_t_*→HL*_t_*_+1_ | *Pgdp*(0.478) *、Tal*(0.239)、 *Str*(0.239) | NT, SX, ZJ, JX, CZh, SH WX, YZ, SZ, NB, CZ, XC MAS, TL, YC, AQ, HF |
| Industry optimizing type | LH*_t_*→HH*_t_*_+1_  HH*_t_*→LH*_t_*_+1_ | *Str*(0.367)、*Pgdp*(0.325) *、Tal*(0.186) | HZ, JH, ZS, TZh |
| Innovation driving type | HL*_t_*→HH*_t_*_+1_  LH*_t_*→LL*_t_*_+1_ | *Tec*(0.343)、 *Str*(0.284) *、Pgdp*(0.273) | NJ, HZh, WH, ChZ |
| Traffic impacting type | HL*_t_*→LH*_t_*_+1_ | *Tra*(0.278)、 *Tec*(0.261)、 *Pgdp*(0.214) | TZ |
